# Supplementary figures and images for: Differential transcript usage in the Parkinson’s disease brain
Source: PLoS Genet. 2020 Nov 2;16(11):e1009182. doi: 10.1371/journal.pgen.1009182 (PMC7660910; doi:10.1371/journal.pgen.1009182)

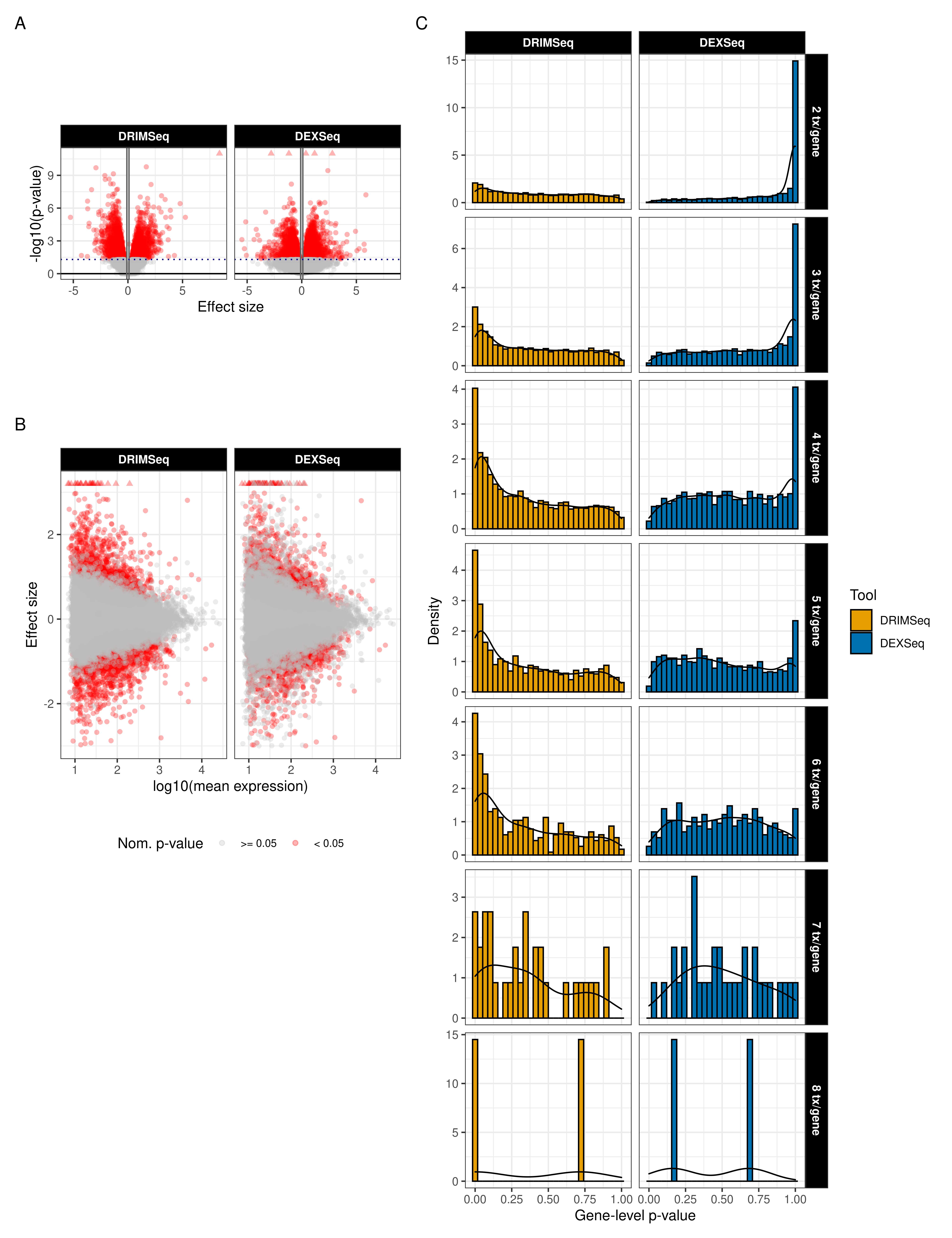

Supplement: S1 Fig — Data points in all plots represent one transcript, with coloring showing significant transcripts (α = 0.05) in red. P-values (uncorrected) are displayed as (−log10(p-value)). A: Volcano plot displaying the effect size (as estimated by the respective tool) in the x-axis and the p-value on the y-axis. Triangles mark extreme p-value outliers that were adjusted to fit into the plot. B: MA plot visualizing a transcript’s significance as a function of its mean expression over all samples. C: Density ridges display the distribution of gene-level significance (−log10(p-value)) per gene type, where genes are grouped according to the number of transcripts they have after filtering. The color gradient was applied to visualize the p-value scale. The vertical dashed line corresponds to a p-value of 0.05. (TIFF) [file pgen.1009182.s001.tiff]

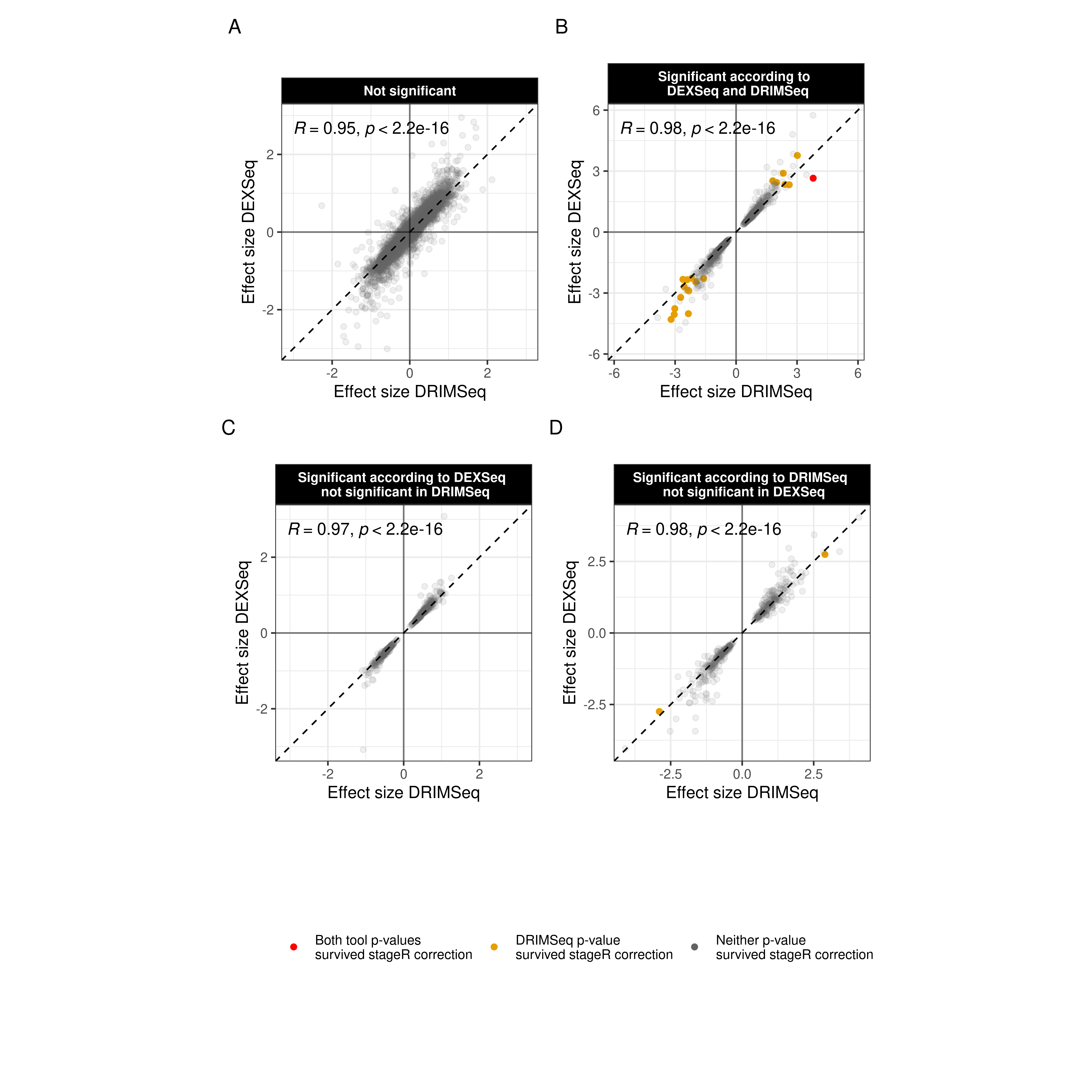

Supplement: S2 Fig — Estimated transcript usage effect sizes are shown for each transcript of the replication cohort, with results from each tool on each of the axes (DRIMSeq x-axis, DEXSeq y-axis). Points situated on the diagonal represent transcripts with equal effect size estimations of both tools; points situated inside the first and third quadrant of the coordinate system represent transcripts agreeing in direction according to both tools (i.e. up-regulated in PD: first quadrant, down-regulated in PD: third quadrant). A: Transcripts that did not reach statistical significance in the DTU analyses by either DRIMSeq or DEXSeq. B Transcripts found to be significant by both tools. C: Transcripts found to be significant by DEXSeq only. D: Transcripts found to be significant by DRIMSeq only. Transcripts identified as DTU events (significant after p-value adjustment) are coloured according to the plot legend. Red: transcript identified as a DTU event by both tools, yellow: transcript identified as a DTU event by DRIMSeq only, grey: transcript either didn’t survive FWER correction by neither tool or wasn’t nominally significant beforehand. (Transcripts can appear significant after FWER control even if they weren’t nominally significant, due to StageR assigning significance by relying on the assumption that if DTU is occurring in the gene (that is: the gene has passed the screening stage) and one of its transcripts is significant, the other must subsequently also take part in the DTU to compensate). (TIFF) [file pgen.1009182.s002.tiff]

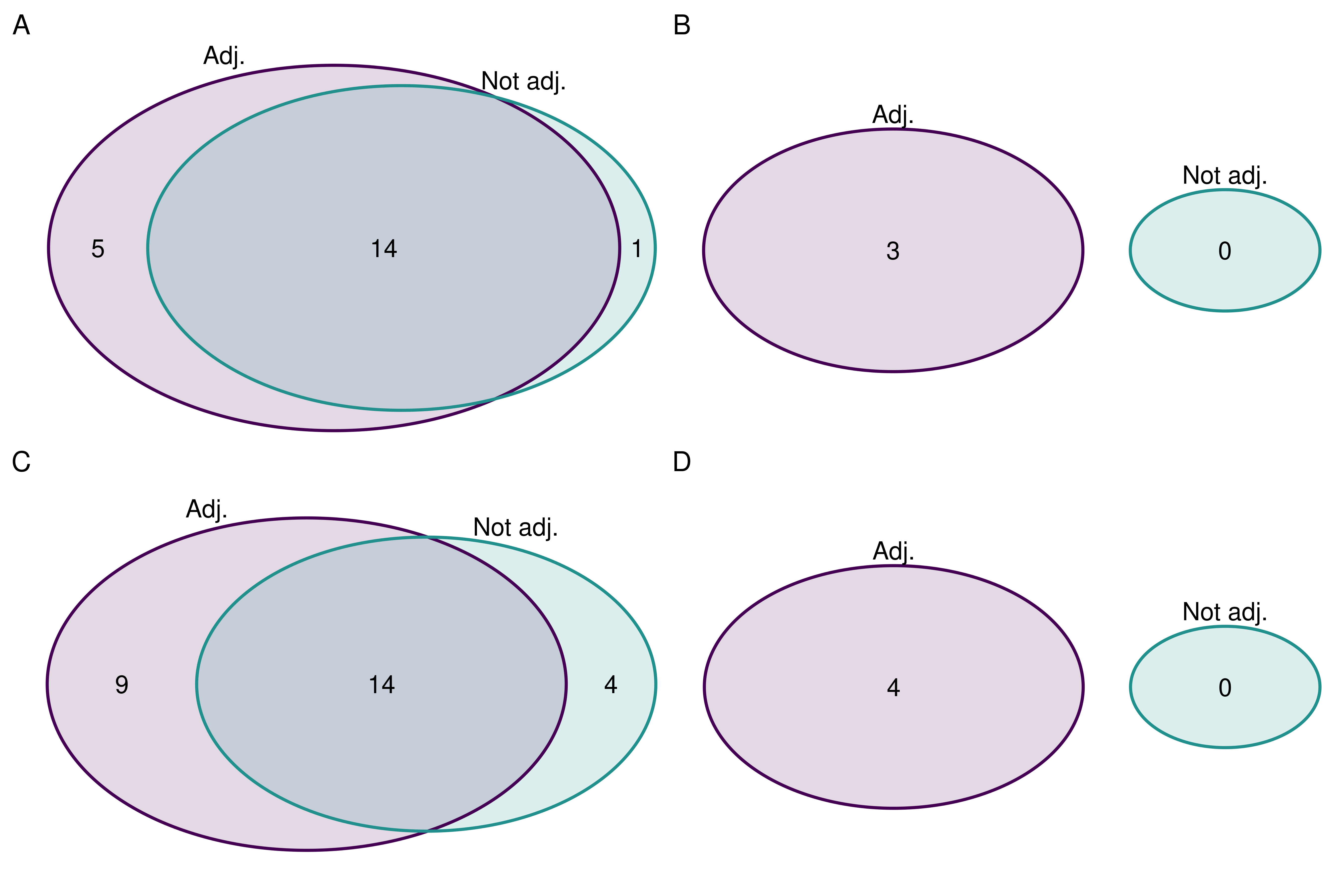

Supplement: S3 Fig — DTU genes (A, B) and events (C, D) resulting from the analysis which included cell type estimations (purple) are overlapped with the results of the analysis where differences in cell types were not taken into account (turquoise). Only DTU events which were identified in the discovery cohort and replicated in the independent replication cohort were considered for this plot. A: DTU genes identified by DRIMSeq. B: DTU genes identified by DEXSeq. C: DTU events identified by DRIMSeq. D: DTU events identified by DEXSeq. (TIFF) [file pgen.1009182.s003.tiff]

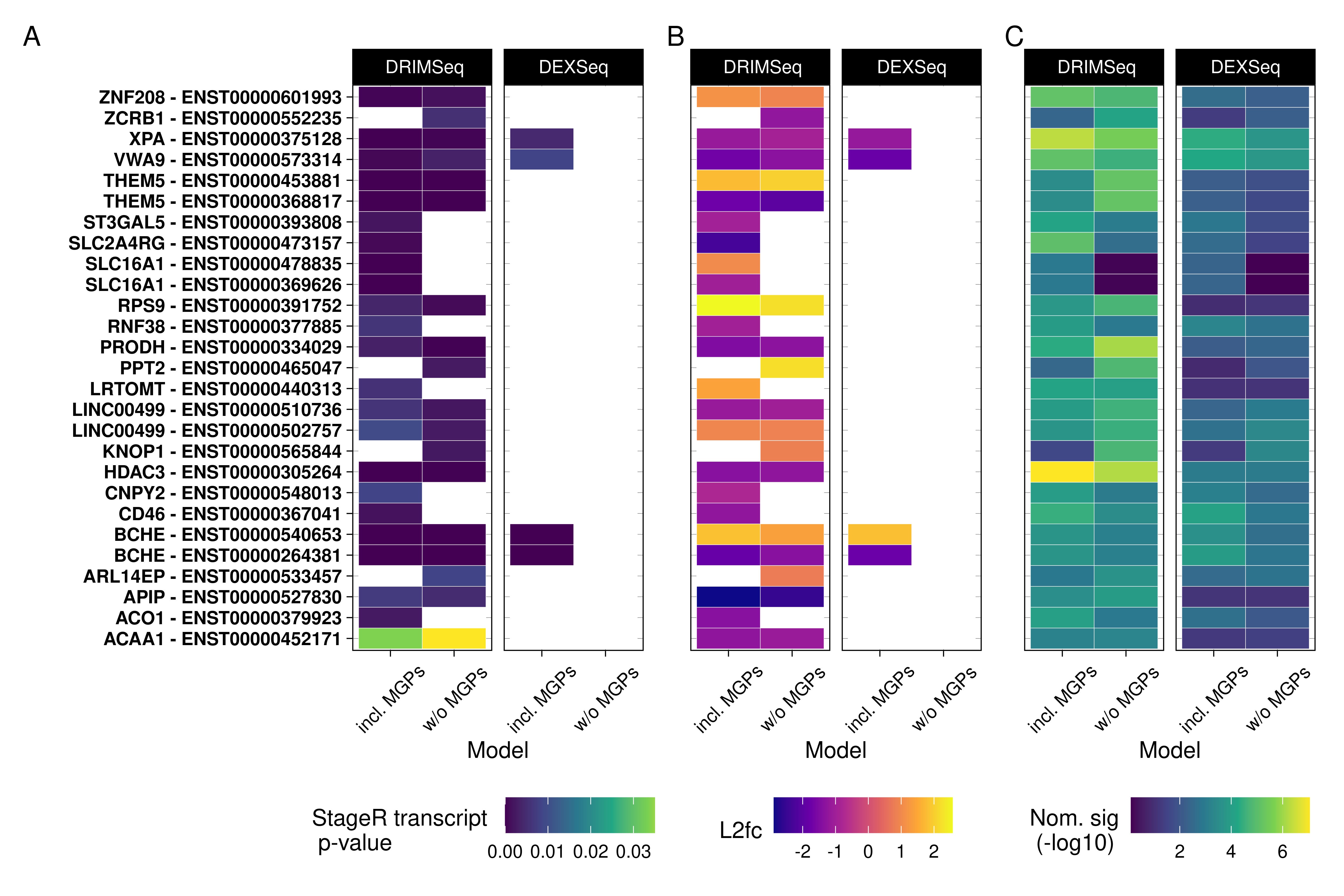

Supplement: S4 Fig — Replicated DTU events (significant after OFWER correction in the discovery cohort, agreeing on the direction of change across cohorts and nominally significant at alpha = 0.05 in the replication cohort) are arranged in the y-axis. A: transcript’s adjusted p-value (white cells indicate adjusted p-value >= 0.05). B: Transcript’s log fold change (white cells correspond to transcripts not identified as DTU events). C: Transcript’s nominal (uncorrected) p-value. In all heatmaps, characteristics are grouped by model design (i.e. with (“Incl. MGPs”) or without (“w/o MGPs”) accounting for MGPs) and by tool (DRIMseq or DEXSeq). (TIFF) [file pgen.1009182.s004.tiff]

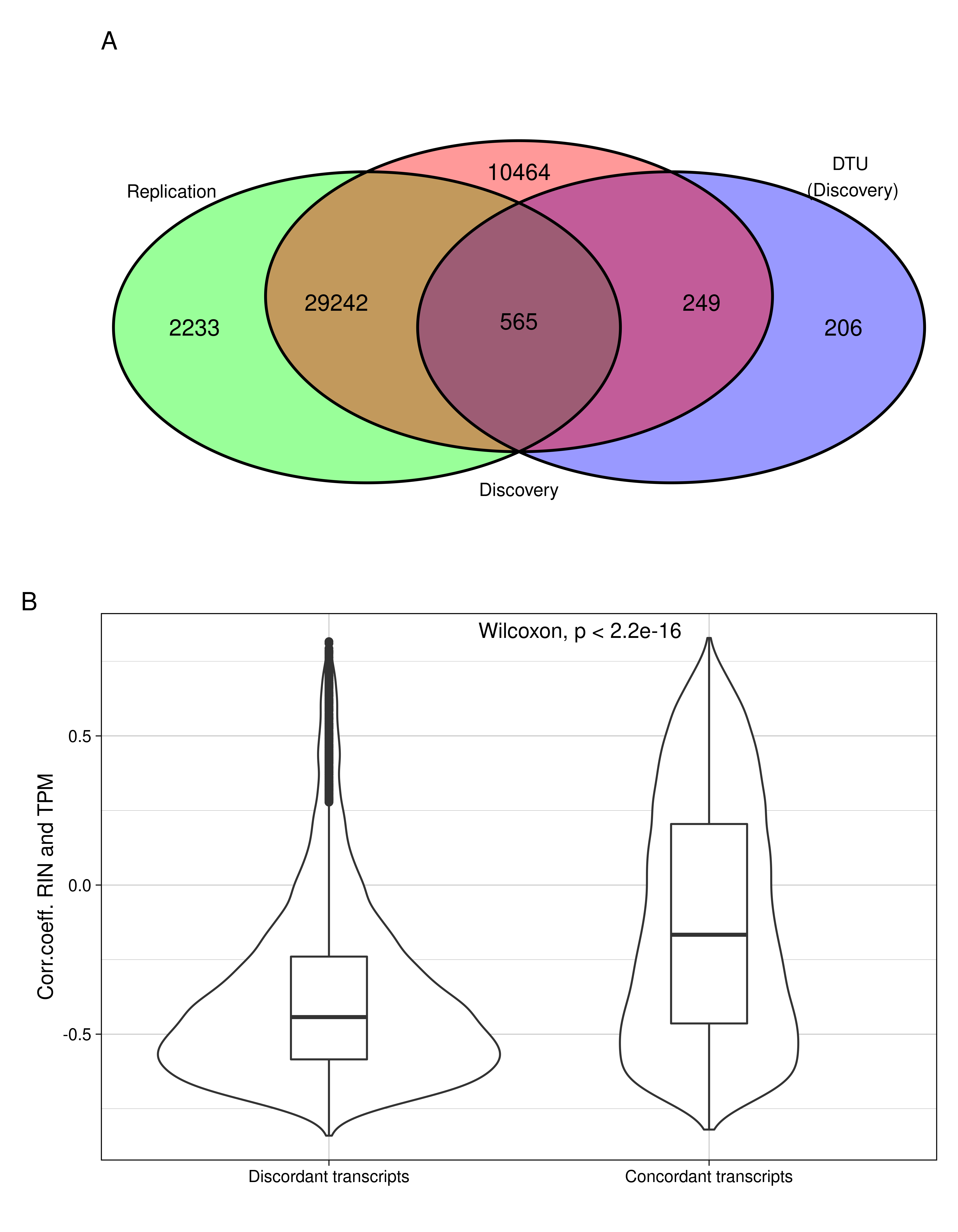

Supplement: S5 Fig — A: Venn diagram for the sets of transcripts which survived pre-filtering in each cohort. Number of transcripts that survived filtering in the replication cohort (green), in the discovery cohort (red), and number of transcripts identified as DTU events in the discovery cohort (blue). B: Distribution of the correlation coefficients between transcript abundance (TPM) and sample RIN for non-concordant transcripts (i.e. transcripts removed during the pre-filtering in the replication cohort, but not in the discovery cohort) and concordant transcripts (i.e. transcripts that survived pre-filtering in both cohorts). (TIFF) [file pgen.1009182.s005.tiff]
