# Supplementary material for: Differential transcript usage in the Parkinson’s disease brain
Source: PLoS Genet. 2020 Nov 2;16(11):e1009182. doi: 10.1371/journal.pgen.1009182 (PMC7660910; doi:10.1371/journal.pgen.1009182)
Supplement: S1 Table — P-values and odds ratios were determined by Fisher’s exact test. The contingency table was built up separating transcripts by whether or not they were identified as DTU events and whether they were defined as the biotype of interest (as defined by Ensembl version 75). The rows are grouped by the tool which identified the DTU event and sorted by increasing p-value of the Fisher’s exact test. (PDF) [file pgen.1009182.s007.pdf]

**Transcript biotype**	**P-value**	**Bonferroni P-value**	**Odds ratio**	**Tool**
Protein coding	1 x 10 ^-10	4x10 ^ -9	0.596	DRIMSeq
Retained intron	3 x 10 ^ -4	0.012	1.552	DRIMSeq
Antisense	7 x 10 ^ -4	0.028	2.233	DRIMSeq
Processed pseudogene	0.001	0.04	0.102	DRIMSeq
Nonsense mediated decay	0.006	0.24	1.859	DRIMSeq
Processed transcript	0.126	1	1.189	DRIMSeq
Unprocessed pseudogene	0.629	1	Inf	DRIMSeq
LincRNA	0.679	1	1.127	DRIMSeq
Unitary pseudogene	1	1	Inf	DRIMSeq
3prime overlapping ncrna	1	1	Inf	DRIMSeq
Misc RNA	1	1	Inf	DRIMSeq
Non stop decay	1	1	Inf	DRIMSeq
Polymorphic pseudogene	1	1	Inf	DRIMSeq
pseudogene	1	1	Inf	DRIMSeq
Sense overlapping	1	1	Inf	DRIMSeq
snoRNA	1	1	Inf	DRIMSeq
snRNA	1	1	Inf	DRIMSeq
Transcribed processed pseudogene	1	1	Inf	DRIMSeq
Transcribed unprocessed pseudogene	1	1	1.256	DRIMSeq
Sense intronic	1	1	Inf	DRIMSeq
Antisense	0.002	0.08	3.363	DEXSeq
Protein coding	0.019	0.76	0.769	DEXSeq
LincRNA	0.028	1	2.532	DEXSeq
processed transcript	0.603	1	0.924	DEXSeq
Transcribed unprocessed pseudogene	0.640	1	Inf	DEXSeq
Nonsense mediated decay	0.701	1	1.169	DEXSeq
Retained intron	0.939	1	1.027	DEXSeq
Polymorphic pseudogene	1	1	Inf	DEXSeq
snoRNA	1	1	Inf	DEXSeq
Unitary pseudogene	1	1	Inf	DEXSeq
3prime overlapping ncrna	1	1	Inf	DEXSeq
non_ top decay	1	1	Inf	DEXSeq
Processed pseudogene	1	1	Inf	DEXSeq
pseudogene	1	1	Inf	DEXSeq
Sense intronic	1	1	Inf	DEXSeq
Sense overlapping	1	1	Inf	DEXSeq
snRNA	1	1	Inf	DEXSeq
Transcribed processed pseudogene	1	1	Inf	DEXSeq
Unprocessed pseudogene	1	1	Inf	DEXSeq
Misc RNA	1	1	Inf	DEXSeq
