# Supplementary material for: Differential transcript usage in the Parkinson’s disease brain
Source: PLoS Genet. 2020 Nov 2;16(11):e1009182. doi: 10.1371/journal.pgen.1009182 (PMC7660910; doi:10.1371/journal.pgen.1009182)
Supplement: Attachment — Submitted filename: DeBoever Review 1.pdf [file pgen.1009182.s008.pdf]

Dick *et al.* present a well-executed study that examines differential transcript usage (DTU) in Parkinson's disease using two independent cohorts. I found the manuscript to be clear and well-written. The methods employed are state-of-the-art and well-supported with references. Some strong points of the paper are the usage of two different approaches for identifying DTUs; usage of a discovery and validation cohort; testing whether cell-type composition affects the results; and the Github repository that provides the code and data necessary for replicating the study. I anticipate that these results will be useful for Parkinson's disease research, especially if the authors can address comment 5 below.

I think the manuscript will be improved by addressing the following comments:

1. Figure 1B: I think the authors should add the frequency of transcript types tested for each transcript type. I would also encourage the authors to include in the figure or in a supplemental table the total numbers of tested and significant transcript types for each of DEXseq and DRIMseq along with an odds ratio and Fisher's exact test p-value (contingency table could be e.g. [[# protein coding sig, # protein coding not sig], [# not protein coding sig, # not protein coding not sig]]) for each transcript type to give a sense of whether DTU events are over- or under-represented in any transcript types.
2. Given the relatively small number of genes represented in the Venn diagrams in S1 Fig. 3, I would suggest instead providing these data in a table or heatmap (where each row is a gene) and indicate which genes were significant in which analyses. Alternatively, the authors could add a supplemental table that shows the DTU summary statistics for these genes in each of the four analyses. This would make it easy to see which specific genes differed between the analyses.
3. Line 168: The authors note that 32,040 transcripts passed filtering in the replication cohort and 93% of those overlapped with the pre-filtered transcripts in the discovery cohort. Can the authors add to the text the number of transcripts that passed pre-filtering in the discovery cohort but not the replication cohort? The authors could also add the information as a Venn diagram in a supplemental figure. It would also be useful to add to the text or supplemental figure the number of DTU transcripts from the discovery cohort that passed filtering in the replication cohort.
4. Line 191: "23% of DTU genes identified in the discovery cohort were filtered out during pre-processing of the replication cohort…" Why is such a large fraction of the DTU genes from the discovery cohort filtered out from the replication cohort? Is it because the replication cohort had a lower sequencing depth per sample or a different reason? If it is due to sequencing depth, perhaps the authors can add a column to S1 Table 1 indicating the total number of mapped or sequenced reads for each sample.
5. I encourage the authors to upload the full transcript/gene count/TPM tables and DTU/DGE summary statistics (e.g. the data provided in S1 Table 2) for all transcripts/genes tested as supporting files or to Figshare, including the cell-type composition and replication analyses. According to the PLOS Genetics Data Availability web site, a Figshare repository will be created automatically for this manuscript, so that might be the best place to upload such files. Please use file formats that can be read by

Excel etc. when file size permits. This would greatly aid in the reusability of the data. The authors have done a fantastic job sharing the code and raw data on Github, but there is a barrier to entry to cloning the repo and getting the R code up and running. Providing count/TPM and summary statistic files on Figshare will allow biologists with less programming experience to interrogate the data and results.
