# Supplementary material for: Differential transcript usage in the Parkinson’s disease brain
Source: PLoS Genet. 2020 Nov 2;16(11):e1009182. doi: 10.1371/journal.pgen.1009182 (PMC7660910; doi:10.1371/journal.pgen.1009182)
Supplement: Attachment — Submitted filename: Response.pdf [file pgen.1009182.s009.pdf]

Dear Prof. Barsh,

Thank you for allowing us to submit a revised version of our manuscript. We are grateful to the reviewers for their comments and have addressed them below point-by-point and in the manuscript. All changes in the manuscript have been marked using *latexdiff*.

Yours sincerely,

Charalampos Tzoulis, MD, PhD
Professor of Neurology and Neurodegeneration
Head, Neuromics Research Group
Head for Neurodegeneration & Vice Center Director, Neuro-SysMed:
Center of Excellence for Clinical Research in Neurological Diseases
Department of Neurology, Haukeland University Hospital
Department of Clinical Medicine, University of Bergen
http://www.neuromics.org/
tzoulis.charalampos@helse-bergen.no
charalampos.tzoulis@nevro.uib.no
Tel: +4755975045
Fax: +4755975164

Reviewer #1:

Dick et al. present a well-executed study that examines differential transcript usage (DTU) in Parkinson's disease using two independent cohorts. I found the manuscript to be clear and well-written. The methods employed are state-of-the-art and well-supported with references. Some strong points of the paper are the usage of two different approaches for identifying DTUs; usage of a discovery and validation cohort; testing whether cell-type composition affects the results; and the Github repository that provides the code and data necessary for replicating the study. I anticipate that these results will be useful for Parkinson's disease research, especially if the authors can address comment 5 below.

Comments:

1. Figure 1B: I think the authors should add the frequency of transcript types tested for each transcript type. I would also encourage the authors to include in the figure or in a supplemental table the total numbers of tested and significant transcript types for each of DEXseq and DRIMseq along with an odds ratio and Fisher's exact test p-value (contingency table could be e.g. [[# protein coding sig, # protein coding not sig], [# not protein coding sig, # not protein coding not sig]]) for each transcript type to give a sense of whether DTU events are over- or under-represented in any transcript types.

We thank the reviewer for the comment and agree that this would improve the clarity of our manuscript. We have included both the frequency of each DTU event in each of the biotype categories and the number of tested transcripts in Fig. 1B, which shows the distribution of identified DTU events per gene across the biotypes. We have also adjusted the figure legend accordingly.

Additionally, we calculated p-values and odds ratios to test for overrepresentation of DTU events in the transcript biotypes using Fisher exact test, as suggested. The code to perform the analysis is updated on github and the table is now included in Supplementary file 2, Table 1. A small paragraph to reference the table was included in the manuscript after referencing Figure 1B (lines 82-88).

2. Given the relatively small number of genes represented in the Venn diagrams in S1 Fig. 3, I would suggest instead providing these data in a table or heatmap (where each row is a gene) and indicate which genes were significant in which analyses. Alternatively, the authors could add a supplemental table that shows the DTU summary statistics for these genes in each of the four analyses. This would make it easy to see which specific genes differed between the analyses.

We followed the reviewer's advice to generate a heatmap that shows the differences between the two analysis models (accounting for cell types and not accounting for cell types). We have added this figure in addition to the Venn diagram and included it in S2 Fig.4.

3. Line 168: The authors note that 32,040 transcripts passed filtering in the replication cohort and 93% of those overlapped with the pre-filtered transcripts in the discovery cohort. Can the authors add to the text the number of transcripts that passed pre-filtering in the discovery cohort but not the replication cohort? The authors could

also add the information as a Venn diagram in a supplemental figure. It would also be useful to add to the text or supplemental figure the number of DTU transcripts from the discovery cohort that passed filtering in the replication cohort.

The number of transcripts which were filtered out in the replication cohort but not in the discovery cohort, and how many of these were identified as DTU events in the discovery cohort, has been added to the main text (lines 182-184). We have also added a Venn diagram to display the overlap of transcripts which survived pre-filtering in S2 Fig.5 A. Please see also our response to comment-4 below (where we further elaborate on this).

4. Line 191: "23% of DTU genes identified in the discovery cohort were filtered out during pre-processing of the replication cohort..." Why is such a large fraction of the DTU genes from the discovery cohort filtered out from the replication cohort? Is it because the replication cohort had a lower sequencing depth per sample or a different reason? If it is due to sequencing depth, perhaps the authors can add a column to S1 Table 1 indicating the total number of mapped or sequenced reads for each sample.

We followed the reviewer's suggestion and added a column to S1 Table 1 ("Library size") with the number of total reads mapped (using Salmon) for each sample. As the reviewer points out, there is a large fraction of DTU genes from the discovery cohort being filtered out from the replication cohort. This is not caused by a lower library-size in the replication cohort (as made evident now in S1 Table 1). However, it is plausible that RNA degradation results in a loss of mapping specificity, increasing the chances of matches to non-primary transcripts. To test this hypothesis, for each of the non-concordant transcripts (i.e. transcripts present in the discovery cohort but filtered out in the replication cohort) we calculated the correlation coefficient between the TPM and RIN. We found, indeed, that for non-concordant transcripts, TPM and RIN had an obvious tendency to be anticorrelated, confirming that lower RNA qualities are associated with more mappings in these transcripts. This anticorrelation is not exhibited by the concordant transcripts (i.e. transcripts that passed pre-filtering in both cohorts. In addition, the non-concordant transcripts have, in their majority, lower TPMs than the non-concordant transcripts (p-value < 2.2 x 10 ^-16, Mann–Whitney U test), further suggesting that they are enriched in unspecific mappings. Considering the overall lower RNA quality of the samples from the discovery cohort compared to the replication cohort, the disparity in terms of filtering statistics is expected. (S2 Fig.5 B). We agree with the reviewer that this is an important point, and we have hence addressed these potential limitations in the discussion of the manuscript (lines 339-347) and included the above results in the additional panel B of S2 Fig. 5.

5. I encourage the authors to upload the full transcript/gene count/TPM tables and DTU/DGE summary statistics (e.g. the data provided in S1 Table 2) for all transcripts/genes tested as supporting files or to Figshare, including the cell-type composition and replication analyses. According to the PLOS Genetics Data Availability web site, a Figshare repository will be created automatically for this manuscript, so that might be the best place to upload such files. Please use file formats that can be read by Excel etc. when file size permits. This would greatly aid in the reusability of the data. The authors have done a fantastic job sharing the code and raw data on Github, but there is a barrier to entry to cloning the repo and getting the R code

up and running. Providing count/TPM and summary statistic files on Figshare will allow biologists with less programming experience to interrogate the data and results.

We have created three tab-separated tables which should be uploaded to the suggested repository in parallel with the submission process:

- *A table that contains sample metadata (including RIN and cell type estimates) together with all covariates used in the analysis*
- *A count matrix table with TPMs as imported with tximport (scaledTPM) for all transcripts and all samples (both cohorts). We have additionally added one column for each cohort to specify whether the transcript passed pre-filtering.*
- *A list containing the result statistics of all identified DTU events (both tools, both cohorts), which we also report as Table 2 in S1.*

Reviewer #2:

It's concise and well-written, with a clear rationale both for the study and the methods used within the study.

From a technical standpoint, they're using tools that model differential transcript usage (DTU) based on transcript counts. While there's certainly an argument in the field as to how accurately these represent differential splicing, the authors are quite conservative in their methodology in that they: 1) follow a pipeline from experts in the field (https://bioconductor.riken.jp/packages/3.10/workflows/vignettes/rnaseqDTU/inst/doc/rnaseqDTU.html#stager-following-dexseq); 2) filter to remove lowly expressed transcript; 3) use two different methodologies (both DTU tools, but based on different underlying distributions) and 4) attempt to replicate in an external dataset. In other words, their methodology is robust.

Other methodological points that I think are worth mentioning/commending the authors for: 1) they correct for cell-type proportions, which is important given that the authors have shown in previous work (Nido et al. 2020) that this confounds gene expression; 2) they attempt to address how isoform changes might impact on disease by looking at effects on subcellular localisation, which I thought was imaginative; 3) the authors have made a very genuine effort to make their analyses transparent and the code reproducible. Looking through their GitHub repository, it is well documented and easy to navigate.

<u>**Comments:**</u>

1. The authors have used GRCh37 and Ensembl v75; the latter was released in December 2013. Why have the authors not used GRCh38 and one of the later versions of Ensembl (which as of April 2020 is now at v100)?

We agree with the reviewer that using the most recent stable version of the reference genome and transcriptome would have been ideal for an entirely novel data series. In this particular case, we were constrained to the use of the GRCh37 assembly (with its most updated corresponding transcriptome), so that our DTU analyses results will be comparable to our previously published work using the same samples, but focusing on differential gene

expression (Nido et al., PMID: 32317022). In that work, RNA-seq was mapped to GRCh37 and we feel it will be an advantage for the readership to be able to make direct comparisons between these different analyses of the same material.

2. First, the authors should be commended on their attempt to validate DTU with qPCR. However, I do have a few questions/comments. The authors test ZNF189 arguing their choice is based on it having i) adequate individual transcript expression levels and ii) sufficiently distinct exonic composition of the individual transcripts to allow transcript-specific amplification. Could the authors expand on these criteria i.e. what was considered "adequate individual transcript expression" and "sufficiently distinct exonic composition"?

We thank the reviewer for the comment and have extended the description of our criteria in the text (lines 100-113). The updated text now explains this in detail:

"To this end, we selected two genes fulfilling the following criteria: i) adequate individual transcript expression levels (i.e., the transcript was present in both cohorts after pre-filtering and detectable by qPCR and ii) sufficiently distinct exonic composition of the individual transcripts to allow transcript-specific amplification (i.e., it was possible to design individual primer pairs that would detect one specific transcript variant alone)."

3. How many DTU genes would have passed the above criteria?

Unfortunately, we are not able to provide an exact number of all DTU genes that would have passed the qPCR criteria. This is because the assessment of each individual gene for qPCR eligibility was carried out manually; namely, by assessing exonic composition and expression for all transcript variants that survived filtering in both cohorts.

As an example, from the list of 19 replicated DTU genes in Table 4, seven genes could have been tested by qPCR for the transcript variants that survived filtering in both cohorts (indicating adequate transcript expression), and were sufficiently distinct in exonic composition (i.e. primer pairs that detect exclusively one transcript variant, but not others, could be designed). However, whether this ratio (~37%) is representative / an approximation for all DTU genes we are not able to determine.

4. Further, it would be helpful for readers if authors provided a figure with the transcript structure of ZNF189 together with the primer sequences used for qPCR to demonstrate how primer choices would ensure transcript-specific amplification.

We thank the reviewer for this comment and have now added a schematic representation of the transcript variants of ZNF189 and the added gene BCHE in the new Figure 2A. The positions of the primers for transcript variant detection are indicated as arrows. The primer sequences are already shown in Table 5, primer sequences for BCHE variants have been added there.

5. I assume that nominal significance is defined as nominal p-value < 0.05, but I could not find any definition of this in the manuscript. It would be helpful to add this.

The reviewer is correct in that nominal significance was defined as a nominal p-value below 0.05. We have added a definition at the end of the methods paragraph that describes the statistical testing (line 499).

6. I could not find any mention of what the read depth of the RNA-sequencing was. It would be useful to add this.

We assume that the reviewer refers to the sample library size. We have added the library size (i.e. sum of aligned reads) to S1 Table 1, as also requested by reviewer # 1. We have also added the target sequencing depth under methods, line 404.

Reviewer #3:

The authors present differential transcript usage in Parkinson's disease (PD) and control brains in a discovery and replication cohort. The study presents novel results regarding the PD transcriptome and contributes to the understanding of disease mechanism. The bioinformatics analysis is rigorous, and the manuscript is well-written.

Comments:

1. The main concern that I have is the lack of concordance between the discovery and replication cohorts. In the discovery cohort, 814 DTU events in 584 DTU genes were found, but only 23 DTU events in 19 genes were nominally significant and concordant in the replication cohort. This might be explained by the heterogeneity in brain tissue samples and the low number of samples used in the discovery and replication cohorts.

We thank the reviewer for this comment. Based on our previous experience and the literature, it is generally not surprising to see low concordance across cohorts in transcriptomic studies conducted in PD brain tissue. As we and others have previously highlighted (e.g. Borrageiro et al., PMID: 29068110, Nido et al., PMID: 32317022), studies of differential gene expression (DGE) show strikingly low replicability at the level of individual genes, although concordance is generally better at the level of pathways.

Our top DTU findings replicate across the two independent cohorts, suggesting these are robustly associated with PD. However, similar to DGE studies, our results show overall low concordance at the gene-level, but converge at the pathway level, where they identify biological processes with established links to PD, such as reactive oxygen species generation and protein homeostasis.

The low concordance observed between the cohorts at the gene level most likely reflects a combination of factors, including: 1) the high biological heterogeneity of PD, 2) the heterogeneous cell-composition of different brain tissue samples, 3) population-specific and technical (brain-bank-specific) differences between the cohorts, 4) differences in RNA quality, sample size and age between the cohorts. We have added a section to the discussion elaborating on these limitations (lines 333-347).

2. The study results would be strengthened by follow-up qPCR analysis in both cohorts of a few of the key genes: THEM5, SLC16A1 and BCHE.

We thank the reviewer for this suggestion. We have carried out further qPCR experiments and added the results for the BCHE transcript variants to the manuscript (lines 110-113) and to the new Figure 2. These results are in full agreement with our RNA-seq based DTU findings for this gene. The exon composition of the SLC16A1 transcript variants did not allow for designing primers that would sufficiently distinguish them from each other. qPCR analyses targeting the two THEM5 variants were carried out, but unfortunately the expression level of variant ENST00000453881 was found too low in several of the investigated tissue samples for robust determination of the variants contribution to overall expression of THEM5. We are confident that the additional data strengthens our findings and are grateful for the possibility to add these data to our manuscript.

3. Also, the lack of replication of previous findings (SNCA, PARK7, PARKN) should be discussed.

We have added a relevant section in the discussion (lines 307-317).

4. The figures are low resolution and hard to read.

We apologize for this inconvenience. It may be that the final compiled document by the submission system resulted in a loss of quality. However, the figures have been submitted as .tiff format in the greatest size allowed by the journal guidelines with a resolution of 300 dpi using lzw compression. We have increased the resolution of the main figures to 600 dpi. We hope that the individual files will be of sufficient resolution.

5. It would be helpful to have the same order of transcript types for DEXSeq and DRIMSeq in Figure 1B.

We adjusted Figure 1B such that in both panels the transcript biotypes are listed in the same order along the y-axis for better comparison.

6. The authors state in methods that "The discovery cohort comprised individuals with idiopathic PD (n = 17) from the Park West study, a prospective population-based cohort which has been described in detail [15] and demographically matched controls (n = 11) from our brain bank for aging and neurodegeneration". Does this mean that the PD and control samples were from different brain banks? I'm assuming that "our brain bank" is the same as Park West but the sentence is confusing and should be reworded.

We agree that the sentence was not formulated precisely and have adjusted it accordingly, such that it becomes clear that the discovery cohort consists of healthy controls and PD from the same brain bank (lines 371-373).
